# A Retrospective Examination of Feline Leukemia Subgroup Characterization: Viral Interference Assays to Deep Sequencing

**DOI:** 10.3390/v10010029

**Published:** 2018-01-10

**Authors:** Elliott S. Chiu, Edward A. Hoover, Sue VandeWoude

**Affiliations:** Department of Microbiology, Immunology, and Pathology, Colorado State University, Fort Collins, CO 80524, USA; elliott.chiu@colostate.edu (E.S.C.); Edward.hoover@colostate.edu (E.A.H.)

**Keywords:** feline leukemia virus, endogenous retroviruses, retroviruses, phylogenetic analysis, subgroup classification, viral interference assays, Sanger sequencing, PCR, next-generation sequencing

## Abstract

Feline leukemia virus (FeLV) was the first feline retrovirus discovered, and is associated with multiple fatal disease syndromes in cats, including lymphoma. The original research conducted on FeLV employed classical virological techniques. As methods have evolved to allow FeLV genetic characterization, investigators have continued to unravel the molecular pathology associated with this fascinating agent. In this review, we discuss how FeLV classification, transmission, and disease-inducing potential have been defined sequentially by viral interference assays, Sanger sequencing, PCR, and next-generation sequencing. In particular, we highlight the influences of endogenous FeLV and host genetics that represent FeLV research opportunities on the near horizon.

## 1. Background

In the early 1960s, William Jarrett described feline leukemia virus (FeLV) as the infectious agent responsible for approximately half of observed cases of feline leukemia and lymphoma [1]. The discovery of this pathogenic gammaretrovirus launched the field of feline retrovirology and discoveries relating to mechanisms of retroviral-induced cancers and oncogenes [2,3]. FeLV was historically a common domestic cat pathogen, and remains one of the few retroviral diseases for which there is an effective vaccine [3,4,5]. As the incidence of FeLV decreased via effective quarantine and vaccination procedures, and with the discovery of feline and simian immunodeficiency viruses as alternate and more analogous models for human immunodeficiency virus (HIV) research, studies of FeLV biology and pathogenesis diminished. Therefore, most of the significant FeLV literature was generated before the development of ‘modern’ molecular techniques. In this retrospective, we review the traditional assays used to establish classical virus subgroups, examine how modern molecular techniques may be used to re-evaluate FeLV subgroup classification schemes, and provide new information to unravel interactions between exogenous and endogenous retroviruses.

## 2. Feline Leukemia Virus Genome Organization

**Figure 1:**
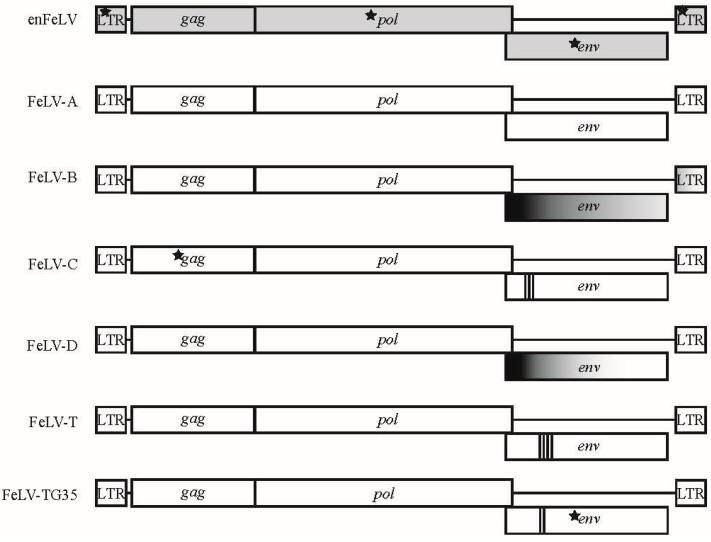
Genomic map of feline leukemia virus (FeLV) subgroups. Six different FeLV subgroups have been associated with different disease outcomes that differ genetically and biologically from endogenous FeLV (enFeLV). EnFeLV is the most genetically distinct from FeLV-A, with nucleotide differences noted in long terminal repeats (LTR), *gag*, and *env*. FeLV-B is formed by recombination of the enFeLV *env*-LTR with FeLV-A. The 5′ recombination site is more conserved than the 3′ site. FeLV-C, T, and TG35 have focal insertions, substitution, and deletions within the parent FeLV-A virus at different regions. Insertions are most often localized to the 5′ *env* and are demarcated here by bold vertical bars, with each line denoting a minimum of one amino acid insertion. Stars denote presence of single nucleotide polymorphisms (SNPs) that are highly concentrated in the respective genes between FeLV-A and other subgroups. FeLV-D displays a recombination event with another domestic cat endogenous virus (ERV-DC; for simplicity, we have not indicated ERV-DC here).

The genome structure of retroviruses includes three genes flanked by un-translated regulatory sequences known as long terminal repeats (LTR). *Gag* encodes group-specific capsid antigens, *pol* encodes protease, integrase, and reverse transcriptase enzymes, and *env* encodes the envelope proteins [6]. FeLV is approximately 8.4-kb in length and lacks accessory genes characteristic of complex feline retroviruses such as feline immunodeficiency virus (FIV) and feline foamy virus (FFV, also referred to as feline spumavirus, FSV). FeLV contains two reading frames, one for *gag* and *pol* genes and a second that encodes the *env* transcript (Figure 1) [3].

## 3. Endogenous Feline Leukemia Virus

As part of the retroviral infection cycle, viral RNA is reverse transcribed into DNA, which enters the nucleus and integrates within the host genome. This process leads to an integrated provirus in host cell DNA, a hallmark of retroviral infection that is a required component of the viral lifecycle. If integration occurs in a germ cell, the provirus can be transmitted vertically through simple Mendelian inheritance [7]. As retrotransposable elements, endogenized retroviruses have duplicate flanking LTRs, and thus can be excised and relocate to other areas of the genome via recombination. Endogenized viruses may acquire mutations that impair productive viral replication, yet remain as endogenous genomic elements fixed in the host genome [8].

Endogenous feline leukemia virus (enFeLV) appears to have invaded the feline genome prior to the speciation of the *Felis* genus [9]. While enFeLVs do not induce disease in the host, they are highly relevant to domestic cat FeLV biology. Endogenous FeLV is expressed in many tissue types and is associated with FeLV infection [10,11,12,13]. Endogenous FeLV integration site and copy numbers vary among individual cats (8–12 copies per haploid genome; up to 19 per diploid genome) due to viral transposition events and multiple independent integrations [8,9,14,15,16]. Increased enFeLV proviral copies have been correlated with both increased [11,12] and decreased [17] susceptibility to FeLV infection, but not with disease progression [11]. Endogenous and exogenous FeLVs (exFeLV) are approximately 86% similar at the nucleotide level. Differences between enFeLV and exFeLV occur in *gag* and *env*, and feature insertions and deletions (INDELs), frameshifts, nonsense mutations, and changes to the unique 3′ regions of the LTR (Figure 1) [3]. As noted below, enFeLV recombination with exFeLV results in novel FeLV subgroups [18], though the relationship between enFeLV and exFeLV infection has not been extensively studied. Because most felid species do not harbor enFeLV, naturally occurring FeLV infections in non-domestic felids provide an opportunity to interrogate protection or promotion of exFeLV by enFeLV in a biologically relevant system.

## 4. Exogenous Feline Leukemia Virus

It is postulated that FeLV arose from a rodent-derived virus that evolved to infect cats as a consequence of predator/prey relationship between cats and mice [2]. Exogenous (horizontally transmissible/infectious) FeLVs have been classified as subgroups, based on functional and genetic relatedness. The first three FeLV subgroups identified (FeLV-A, B and C) were characterized using viral interference (VI) assays, and eventually were associated with subgroup-specific clinical phenotypes [19,20,21]. Definition of FeLV subgroups by Oswald Jarrett et al. was an early area of intense FeLV study because of their relation to differences in disease progression and prognosis. FeLV-A is the most common horizontally transmitted subgroup [22,23]. While FeLV-A has been reported to be less pathogenic than other FeLV subgroups, it has been associated with macrocytic anemia, immunosuppression, and lymphoma [3,24]. FeLV-B, a recombinant of FeLV-A with enFeLV, has been reported to occur in approximately half of cats infected with FeLV-A. It arises by recombination between FeLV-A and enFeLV subsequent to co-packaging of expressed enFeLV and exFeLV transcripts into a single virion, followed by strand displacement during reverse transcription [25,26,27]. FeLV-B is tumorigenic [24], and is considered to be incapable of horizontal transmission unless it is co-transmitted with FeLV-A [20], with rare exception [28,29]. FeLV-C is a less common subgroup that arises from de novo mutations in *env* of FeLV-A and has been associated with the development of aplastic anemia [3,30,31,32,33,34,35].

## 5. Viral Interference Assays

Viral interference (VI) assays test the ability of one viral strain to limit infection with a second viral isolate. Viral interference occurs via both intrinsic and extrinsic mechanisms resulting from cellular pathways that are perturbed during viral infection. Extrinsic VI is caused by competitive blockage of virus receptor by proteins or other viruses that bind and occlude receptor-mediated entry for subsequent viruses. Intrinsic VI refers to multiple processes including intra-cellular receptor fatigue [10,36,37,38], interferon-mediated interference in response to viral genetic material [39], and superinfection exclusion [40].

**Figure 2:**
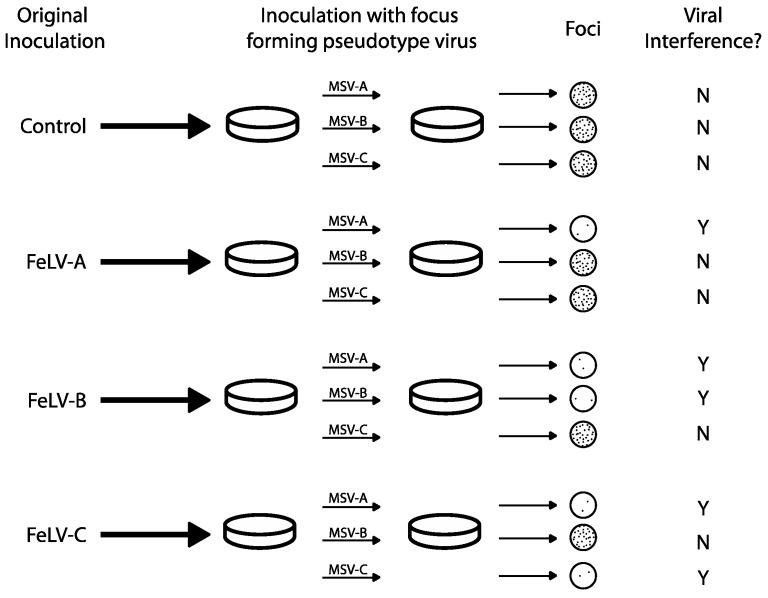
Predicted outcomes of FeLV viral interference as defined by Sarma and Log, 1971 [19]. FeLV subgroups were first identified by ability or inability of virus types to infect Murine sarcoma virus (MSV)-infected hamster cells (Sarma and Log, 1971 [19]). Focus-forming pseudotypes (chimeras with the ability to form plaques) were plated on previously infected cell cultures. Cultures with a 2-log reduction in focus-forming units were considered to demonstrate viral interference.

Viral interference assays were used to distinguish and initially define FeLV subgroups A, B and C, presumably via intrinsic mechanisms. FeLV viruses that “interfere” with one another (i.e., virus A precludes superinfection with virus B) were tested by a classical method to identify viral groups of the same subgroup (which interfere) versus viruses of different subgroups (which do not interfere) [41,42]. In 1971, Sarma and Log used interference assays to establish the first three recognized FeLV subgroups: A, B and C (Figure 2) [19]. Focus-forming FeLV/murine sarcoma virus (MSV) pseudotypes (viral chimeric constructs in which MSV envelope proteins have been replaced by FeLV *env*) were produced by rescue of 9 natural tumorigenic FeLV isolates following co-culture on Harvey MSV-infected hamster tumor cells and feline embryonic fibroblasts. Subsequent in vitro infection of feline embryo fibroblasts with one subgroup resulted in the blockage of the corresponding pseudotype. Cell cultures were considered to demonstrate viral interference if a 2-log drop in focus forming titer was measured. For example, when feline embryo fibroblast cultures were infected with FeLV-A, they were still susceptible to FeLV-B and C pseudotypes (i.e., foci were present following secondary infection). Additionally, cells infected with FeLV-C were susceptible to FeLV-B pseudotype infection, and vice versa (Figure 2). These experiments led to the conclusion that FeLV-A, B and C were genetically different and capable of superinfection in cells.

Curiously, primary infection with FeLV-B or FeLV-C virus blocked subsequent infection of FeLV-A pseudotype. This unexpected display of viral interference between different strains subgroups provided evidence for co-infection between FeLV-A and other. This led to the hypothesis that FeLV-A is a necessary precursor for the development of more pathogenic FeLV subgroups and is an essential helper virus for other subgroups. Subgroups were further described by demonstrating that neutralizing antibodies raised in goats and cats inoculated with different strains demonstrated subgroup neutralizing specificity, further elucidating variation among subgroups [43]. Using this criterion, FeLV-A was more monotypic compared to FeLV-B and C, which displayed more antigenic variation.

On a functional level, VI among FeLV subgroups may be explained by variation in receptor use (extrinsic interference). FeLV-A uses thiamine transporter receptors (ThTR-1) [44] while FeLV-B uses a common retroviral entry receptor, the phosphate transporter receptors (PiT-1/2) [45,46,47,48]. FeLV-A env would bind ThTR-1, which would not preclude binding to PiT-1/2, but cells infected with FeLV-B would not be permissive to an additional FeLV-A infection as FeLV-B infections almost always involve a FeLV-A co-infection. FeLV-C uses a heme exporter receptor (FLVCR-1/2) along with ThTR-1/2 [49,50,51].

## 6. Sanger Sequencing

Now a fundamental technique in molecular biology, Sanger sequencing was developed in 1977, after FeLV was discovered and classified by VI assays [19,20,52]. Sanger sequencing introduced nucleotide analysis allowing researchers to understand and associate FeLV genetic sequences with functional proteins [53]. Additionally, other FeLV subgroups marked by relatively minor genetic variations were identified, making subgroup identification more complicated.

**Figure 3:**
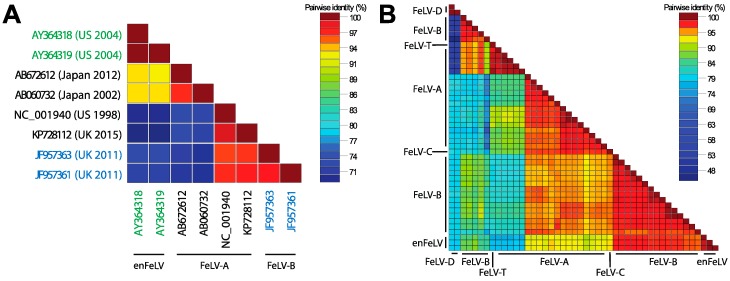
Pairwise identity across FeLV subytpes. (**A**) Full genomes of enFeLV (green font), FeLV-A (black font), and FeLV-B (blue font) document discrimination of two major groups (indicated by blue/green grid and yellow/red grid). Pairwise identify is indicated by color scale of intersecting grid blocks. FeLV-A is highly conserved (>94% pairwise identity), though two subgroups are indicated by red versus yellow-orange grid colors. Isolates demonstrate great variation between clades (70–77% pairwise identity with highest conservation in *gag* and *pol*), although genetic similarity is not entirely driven by subgroup; (**B**) pairwise identity of the *env* genes demonstrates that this region is most divergent among FeLV subgroups. Sequence accession numbers used for analysis: enFeLV—AY364318-9, M25425; FeLV-A—AB060732, AB635483, AB635500, AB635510, AB635516, AB672612, EU359303-6, EU359308-9, KP728112, M12500, M18247-8, M89997; FeLV-B—AB635492, AB635494, AB635499, AB635502, AB635506, AB635512, AB635517, AB635526, AB635579, AB635581, AB635638, AF403716, J03448, JF957361, JF957363 K01208-9, V01172, X00188; FeLV-C—M14331; FeLV-D—AB673426, AB673432; FeLV-T—M18246.

In 1980, Rosenberg et al. conducted a sequence-level comparative analysis of FeLV-A, B and C. Homology indices based on 2D polyacrylamide gel electrophoresis (PAGE) fingerprinting were low among all subgroups (37–66%) [54]. Modern sequencing technologies have allowed full genome analyses of FeLV, and documented homology among all subgroups and enFeLV by pairwise comparison. Figure 3 illustrates strain similarities using SDTv1.2 nucleotide pairwise comparison tool following Multiple Alignment using Fast Fourier Transform (MAFFT) [55]. FeLV-A displays the strongest sequence conservation among distinct FeLV-A isolates, with some genes having 98% homology [56,57]. Other subgroups are less well conserved. For instance, FeLV-B was first characterized as having up to ten variable regions with respect to FeLV-A [26,33,58,59]. The sequences of the variable region depend on the enFeLV source. enFeLVs have not been rigorously examined at the nucleotide level; as a result few FeLV-B sequences have been recorded to allow for detailed nucleotide comparisons [25,33,53,58,59,60,61,62,63,64,65]. Variable regions 1–5 (*vr1*–*5*) and potentially the *C*-terminus domain are believed to be responsible for altering cellular tropism due to changes in the receptor binding protein (gp70) based on phylogenetic analysis [25,59,66]. Few studies have been performed to document consequences of amino acid variation in other variable regions. Alignment and comparative analyses of enFeLV, FeLV-A and FeLV-B sequences identify a relatively conserved 5’ recombination site in the 5′ gp70 gene. A 3′ recombination site region is also evident, but is more variable [27,57,67]. Variation in recombination sites between enFeLV and exFeLV results in nucleotide divergence among FeLV-B genotypes, particularly in the envelope gene. However, FeLV-B’s still share significant pairwise identity to the closely related FeLV-A’s. Work from the laboratories of Roy-Burman and Overbaugh examining exFeLV/enFeLV recombination during in vitro infections has revealed that replication efficiency and cellular tropism depends on the length and region of the enFeLV sequence incorporated into the FeLV-B recombinant [68]. Amino acid changes localized to two variable regions (VRA and VRB) mediate the ability of FeLV-B to bind to receptors Pit1 and/or Pit 2 [60,61]. Aside from changes to the *env* gene, FeLV-B recombinants have been described that incorporate enFeLV sequences in the LTR region and *gag* gene [69]. Curiously, while enFeLV is seen as a necessary progenitor for the generation of FeLV-B, it has also been posited that truncated enFeLV Env may act to interfere with FeLV-B infection [10].

Sequence analysis of FeLV-C linked genotypic determinants to disease phenotypes [33]. Changes in the FeLV-C 3′ *pol* and 5′ *env* gene are associated with aplastic anemia and expand the host range to other species in cell culture [59]. Naturally occurring FeLV-C isolates demonstrate that FeLV-C is the result of amino acid changes in the N-terminal portion of the surface protein. Further studies indicated that an 886-bp fragment from FeLV-C encompassing the 3′ end of *pol* (73 amino acids) and the 5′ end of *env* (241 amino acids) to a recipient FeLV-A were necessary to confer the fatal aplastic anemia phenotype [59]. Subsequent analysis indicated that a three-codon deletion within the first variable region of the *vr1* of the 5′ *env* gene and nine adjacent substitutions may be sufficient to confer virulent phenotype [62,63]. These findings suggest precise mutations at specific loci may dictate disease phenotypes typically ascribed to FeLV-C.

FeLV-61C (aka FeLV-T), is a T-cytopathic FeLV subgroup capable of forming syncytia in 3201 cells, was first isolated in a natural thymic lymphoma [70,71]. FeLV-T induces a fatal immunosuppressive disorder described as FeLV-FAIDS (feline acquired immune deficiency syndrome) [72]. The subgroup was characterized following experimental infections of a domestic cats with a transmissible FeLV clone, 61E [35,72,73]. An infected cat subsequently developed thymic lymphoma, atypical of FeLV-A infection, and tissues were analyzed for mutations underlying this phenotype [35]. Sequence analysis revealed a variant of primary FeLV-A *env* containing a 6-amino acid insertion and 6-amino acid deletion [71]. Another FeLV variant with a 4-amino acid insertion, (81T), was shown to be sufficient to induce the FeLV-T phenotype [65,74]. This variant, like 61C, was replication-defective [75]. Chimeras generated from 61E and 81T generated tissue culture-adapted isolates with compensatory mutations at positions 7 and 375, rescuing the Env processing ability. These changes both occur outside of the receptor-binding domain [75]. Further research documented that FeLV-T is incapable of membrane fusion to its receptor (Pit1) due to a histidine-aspartate substitution at the N-terminus [76]. Infection is possible only in the presence of FeLIX, a truncated envelope protein constitutively produced by enFeLV, which shares greater than 90% identity to FeLV-B *env* [77]. Ultimately, the progressive FeLV-FAIDS disease progression and augmented cellular tropism led to classification the FeLV-T subgroup [65,71].

In the late 1980s, Levesque et al. examined naturally occurring FeLV from a group of cats experiencing lymphomas. One animal had developed a multicentric lymphoma that was non-B-cell non-T-cell in origin [78]. LTR recombinants of FeLV-945 and a closely related retrovirus, Moloney murine leukemia virus, were identified in tumor tissue [79]. Variant FeLV-945 was shown to have a specific 21-bp tandem triplication repeatedly identified in independent multicentric lymphomas, conferring a replicative advantage in feline cells [80,81,82].

The two most recent additions to the FeLV subgroup family include the less characterized FeLV subgroups D and TG35. FeLV-D was identified concurrent with the discovery of a novel domestic cat endogenous retrovirus (ERV-DC) that is divergent from enFeLV [83]. Transduction of the ERV-DC *env* gene into FeLV produced FeLV-D that displayed novel receptor interference patterns [84]. As has been hypothesized with FeLV-B, FeLV-D appears to be restricted by an ERV-DC envelope-like antiretroviral factor termed, Refrex-1 [84,85]. FeLV-TG35 was identified in a 1-year-old castrated male cat. One of several *env* clones (TG35-2) harbored a seven amino acid substitution and two amino acid insertions in the *vr1*. Although the sequence bore resemblance to FeLV-A, interference assays confirmed that TG35-2 Env targeted a different receptor, potentially constituting a new subgroup [64].

This review of novel FeLV variants and subgroups demonstrates a wide range of sequence heterogeneity. Some subgroups represent infrequent point mutations, while others represent recombination events resulting in substitution of nearly 30% of exFeLV genome. Determining whether particular isolates are new subgroups vs. variants is reminiscent of the splitter-lumper debates that are innate to taxonomy, systematics, and nosology [86,87,88,89]. Since not all FeLV sequenced variants have been definitively associated with disease, it is also unclear if some sequenced isolates represent truncated defective viruses that are apathogenic. The presence of variable enFeLV proviral copy number and genotype provide a rich potential for the generation of many new FeLV variants during the course of infection.

## 7. Polymerase Chain Reaction

The development of PCR in the early 1980s provided scientists with the ability to directly target specific nucleic acid sequences for amplification and detection, either by visual or digital optic means [90]. The specificity of a PCR assay depends on the primers used to discriminate between targets. The presence of enFeLV in all domestic cats has added additional challenges to the understanding of FeLV biology. EnFeLV can exist as nearly full length pseudogenomes or may be present as a small fraction of the genome consisting solo LTRs. This factor, coupled with the significant homology between enFeLV and exFeLV genomes makes PCR differentiation of these two forms challenging. Regions of relatively high sequence heterogeneity in the *env* gene and LTR sequences have been exploited to develop PCR primer targets to distinguish between enFeLV and exFeLV subgroups [9,91]. This has allowed investigators to begin to interrogate interactions between the enFeLV genotype and exFeLV susceptibility and disease outcome [12]. Additionally, infection outcome categories previously characterized by antigen detection have been re-examined using PCR focusing on the differences in proviral load and viremia [91,92,93]. This advance allowed for the definition of 4 different viral outcomes based on viral load: progressive, regressive, latent, and abortive [24,91,93]. Determination of proviral and viral loads and correlation with FeLV subgroups and tissue tropism will further help to understand determinants of FeLV pathogenesis.

## 8. Next Generation Sequencing and Beyond

Viral infections typically result in populations of viral quasi-species representing a vast amount of diversity [94]. Despite its relatively slow mutation rate compared to other retroviruses, FeLV can be detected within a single infected cat as a population of multiple variants [95]. Next generation sequencing (NGS) can be implemented in FeLV research to examine within host or within population viral diversity, enFeLV and exFeLV integration sites, and physiological responses to infection, which have formerly been inferred using indirect genetic analysis [96,97,98,99]. As of this writing, only one group has used NGS as a methodology for examining FeLV [13]. In this RNA-seq study, Krunic et al. measured a 3.4-fold decrease in enFeLV expression in feline lymphomas compared to case controls. NGS methods could allow for re-examination of enFeLV infection interactions in the presence and absence of exFeLV [100,101].

While NGS will open a new frontier for FeLV studies, significant challenges are inherent in enFeLV genotypic analysis. Assembling exogenous FeLV provirus are complicated by the presence of enFeLV, given the high homology between the two forms of the virus and variation in insertion sites within the feline genome. These difficulties will likely be overcome by rapidly occurring advances in analytic analyses.

## 9. Concluding Remarks

The history and biology of FeLV infection has been enriched with the introduction of modern sequencing methods. Multiple FeLV subgroups and the virus’ propensity to interact with endogenous elements of the feline genome provide unique viral replication and transmission attributes that significantly impact disease outcomes. Viral interference assays initially determined the virus biological activity and co-infection profiles of FeLV, and predicted genomic changes that were discovered years later with remarkable precision. Sanger sequencing has allowed partial resolution of genotypic characterization of subgroups, resulting in a greater understanding of FeLV diversity. PCR has allowed further dissection of viral replication kinetics during infection, and next generation sequencing provides a future landscape to derive additional information about this interesting virus and its interaction with host genomic elements. Despite the introduction of each new technology, classical techniques continue to identify historic and novel subgroups. Questions that remain include: How should we regard FeLV subgroups, and how should they be classified? Should a minimum sequence length or disease outcome define FeLV subgroups, or should subgroups be defined based on cellular tropism and demonstrated ability to replicate in vitro or in vivo? What are the molecular mechanisms and genotypic correlates that underlie disease phenotypes and outcomes? How does enFeLV genotype influence exFeLV susceptibility and disease outcome? This unique virus will continue to be an important pathogen to both domestic and wild felids. Naturally occurring infections can provide an interesting basis for examination of interactions between endogenous elements and exogenous viral agents in mammalian hosts.
